# Schwerstverletztenversorgung mit aktiver Blutung unter Apixaban und gerinnungsrelevanter Restaktivität

**DOI:** 10.1007/s00113-023-01310-3

**Published:** 2023-03-28

**Authors:** Marc Maegele, Benedikt Stolz, Stefan Trojan, Igor Lazarevits, Martin Linde, Jürgen Meyer-Zillekens, Mathias Formesyn, Eliza von der Leyen, Ulrich Limper

**Affiliations:** 1 Klinik für Orthopädie, Unfallchirurgie und Sporttraumatologie, Kliniken der Stadt Köln-Merheim, Universität Witten/Herdecke (UW/H), Campus Köln-Merheim, Ostmerheimerstr. 200, 51109 Köln, Deutschland; 2 Institut für Forschung in der Operativen Medizin (IFOM), Universität Witten/Herdecke (UW/H), Campus Köln-Merheim, Köln, Deutschland; 3 Klinik für Anästhesiologie und Intensivmedizin, Kliniken der Stadt Köln-Merheim, Universität Witten/Herdecke (UW/H), Campus Köln-Merheim, Köln, Deutschland; 4 Klinik für Viszeral‑, Tumor‑, Transplantations- und Gefäßchirurgie, Kliniken der Stadt Köln-Merheim, Universität Witten/Herdecke (UW/H), Campus Köln-Merheim, Köln, Deutschland; 5 Institut für Luft- und Raumfahrtmedizin, Deutsches Zentrum für Luft- und Raumfahrt (DLR), Köln, Deutschland

**Keywords:** DOAK, Trauma, Blutung, Diagnostik, Antagonisierung, DOAC, Trauma, Bleeding, Diagnostics, Reversal

## Abstract

Direkte orale Antikoagulanzien (DOAK) werden zunehmend zur Prophylaxe thrombembolischer Ereignisse eingesetzt. Ihr Umgang, insbesondere in Notfallsituationen, gestaltet sich schwierig, da Spiegelmessungen oft nicht zeitnah zur Verfügung stehen und bis vor Kurzem keine Möglichkeit zur Antagonisierung bestand. Es wird die notfallmäßige Behandlung eines Schwerstmehrfachverletzten mit lebensbedrohlicher traumatischer Blutung unter Dauertherapie mit dem Faktor-Xa-Hemmer Apixaban, viskoelastizitätsbasierter Detektion gerinnungsrelevanter Restaktivität bei Schockraumaufnahme und gezielter Wirkungsaufhebung beschrieben.

## Abstract (en)

Direct oral anticoagulants (DOAC) are increasingly being prescribed for prophylaxis of thromboembolic events. Their use, particularly in emergency settings is difficult as blood level measurements are often not immediately available and until recently there was no possibility for reversal. This article presents the case of a severely injured patient with life-threatening traumatic bleeding under long-term treatment with the factor Xa inhibitor apixaban, viscoelasticity-based detection of residual systemic anticoagulatory activity and targeted reversal.

Direkte orale Antikoagulanzien (DOAK) haben Phenprocoumon zur Prophylaxe thrombembolischer Ereignisse weitestgehend abgelöst, und ihr klinischer Gebrauch wird weiter zunehmen. Während es für die Elektivchirurgie Handlungsempfehlungen zum sicheren Umgang mit diesen Substanzen gibt, fehlen diese bislang für die Akutsituation mit der Notwendigkeit für einen chirurgischen Notfalleingriff. Inzwischen existieren spezifische Antidote und neue Messmethoden zur besseren Handhabung von DOAK in der unfallchirurgischen Notfallsituation.

## Falldarstellung

### Anamnese: Prähospitale Versorgung

Die Aufnahme des 54-jährigen, männlichen Patienten erfolgte luftgebunden nach einem Verkehrsunfall mit Einklemmung und technischer Rettung. Bei Eintreffen des Rettungsdienstes war der Patient wach und ansprechbar (Glasgow Coma Scale [GCS] 14) mit Schmerzen abdominell und in beiden unteren Extremitäten. Bei klinischem Verdacht auf eine geschlossene Unterschenkelfraktur rechts erfolgte die Schienung, bei ebenfalls klinischem Verdacht auf eine Beckenfraktur mit proximaler Mehretagenfraktur des linken Femurs die Anlage einer Beckenschlinge. Kardiothorakopulmonal imponierte die Situation insgesamt kompensiert bei ultrasonographischem Nachweis eines Flüssigkeitssaums zwischen Milz und linker Niere (Koller-Pouch) und klinisch leichter Abwehrspannung. Auf Befragen des Notarztes bejahte der Patient die dauerhafte Einnahme des Faktor-Xa-Hemmers Apixaban. Nach Anlage von 2 großlumigen Zugängen zur restriktiven Volumentherapie erfolgte unter Analgosedierung sowie axialer Stabilisierung auf der Vakuummatratze der Transport in die Zielklinik.

### Befund: Versorgung und Diagnostik im Schockraum

**Figure 1:**
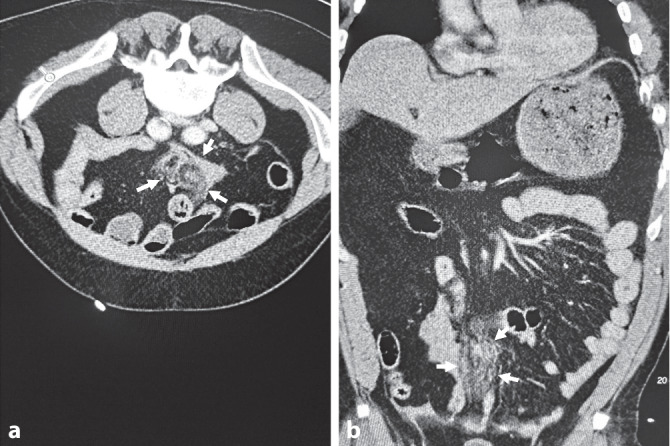

Bei Aufnahme im Schockraum der Zielklinik zeigte sich ein tachykarder (Herzfrequenz 125/min), normotoner (mittlerer arterieller Blutdruck [MAP] 100 mm Hg) und analgosedierter Patient (GCS 11) mit einer Spontanatemfrequenz von 20/min unter 10 l/min Sauerstoff. Der intraabdominelle Flüssigkeitsnachweis um Milz und Blase wurde im Schockraum ultrasonographisch bestätigt und in Rücksprache mit dem Notarzt als größenzunehmend gewertet. Es erfolgte die Gabe von 1 g Tranexamsäure intravenös. Laborchemisch zeigte sich ein Hb 15,6 g/dl bei einem pH 7,269, Lactat 4,4 mmol/l und BE −1,1 mmol/l; als Zeichen des Abdominaltraumas waren die Leberenzyme leicht erhöht, und es zeigte sich ein Kreatinin von 1,62 mg/dl als Zeichen einer eingeschränkten Nierenfunktion. Im Rahmen der konventionellen Gerinnungsdiagnostik imponierten eine International Normalized Ratio (INR) 2,75, PTT 38,4 s, Thrombozyten 204/nl und D‑Dimere 18,7 mg/l. Die computertomographische Ganzkörperschnittbildgebung zeigte als intraabdominelle und aktive Blutungsquellen mehrere Mesoeinrisse (Abb. 1); das weitere Verletzungsmuster bestand aus multiplen Lungenkontusionen, einer Dreietagenfraktur des linken Femurs mit begleitender drittgradig offener Fraktur der Patella, einer geschlossenen Unterschenkelfraktur rechts sowie einer Dissektion der linken A. iliaca communis.

### Therapie und Verlauf: „Point-of-care“-Diagnostik und Gabe des Antidots

**Figure 2:**
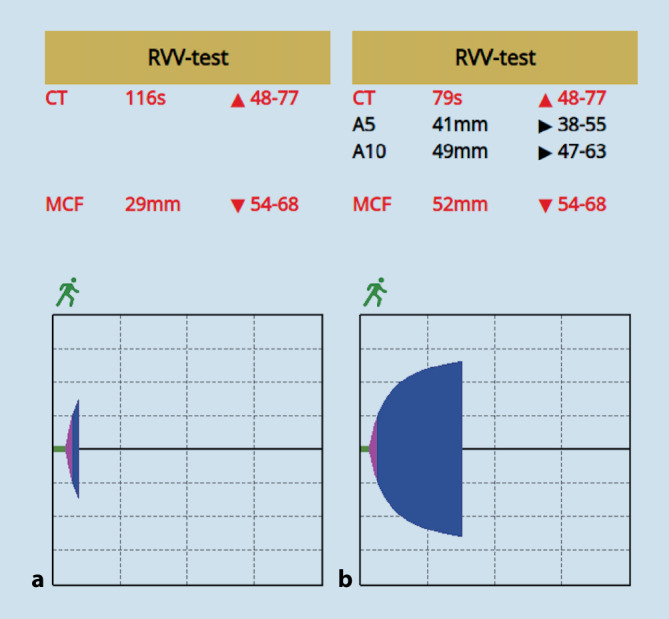

Bei zunehmender kardiozirkulatorischer Dekompensation mit hämodynamisch relevantem Zirkulationsproblem (C‑Problem) infolge der aktiven Blutung wurde der Patient zur notfallmäßigen Laparotomie in den OP verbracht. Bei eigenanamnestischer Dauereinnahme von Apixaban erfolgte die „Point-of-Care“ viskoelastizitäts-basierte 6‑Kanal-Gerinnungsdiagnostik mittels ClotPro® (Fa. enicor GmbH, München, Deutschland). Hier zeigte sich nach 4‑minütiger Laufzeit im Russell-Viper-Venom-Test (RVV) eine Verlängerung der CT-Zeit auf 116 s als Hinweis auf eine noch deutlich gerinnungsrelevante und systemische Apixabanrestaktivität (Abb. 2); die Gerinnungszeit im Test zur Einschätzung des Gesamtgerinnungsprozesses (EX-Test) war auf 69 s verlängert. In der Zusammenschau aus gesichert klinisch-relevanter und aktiver intraabdomineller Blutung mit progredienter lebensbedrohlicher Schocksymptomatik, gerinnungsaktiver Apixabanrestaktivität sowie Verdacht auf eine eingeschränkte Apixaban-Clearance durch die laborchemisch erhobene Niereninsuffizienz erfolgte im interdisziplinären Konsens die Antagonisierung mittels Andexanet alfa. Bei vermutet letztmaliger Einnahme von Apixaban in der Standarddosierung von 2,5 mg am Morgen und damit im Zeitfenster < 8 h zum aktuellen Zeitpunkt erfolgte die Gabe von Andexanet alfa in der niedrigen Dosierung als i.v.-Bolusgabe von 400 mg mit einer Infusionsgeschwindigkeit von 30 mg/min über 15 min, gefolgt von einer Dauerinfusion über 120 min mit einer Infusionsgeschwindigkeit von 4 mg/min (480 mg) gemäß den Herstellerempfehlungen. Im Intervall erfolgte die Wiederholung der Gerinnungsdiagnostik mittels ClotPro® mit Normalisierung der RVV-CT-Zeit (Abb. 2). Zur Stabilisierung der Gesamtgerinnungsfunktion erhielt der Patient bei initial erhöhter INR und verlängerter ClotPro®-EX-Zeit insgesamt 2000 IE Prothrombinkomplexkonzentrat (PPSB) sowie 4 Frischplasmakonzentrate. Parallel hierzu erfolgten die chirurgische Blutstillung und die Versorgung der Frakturen nach dem „Damage-Control“-Prinzip.

Nach Antagonisierung und aggressiver Stabilisierung der Gesamtgerinnungsfunktion wurde intraoperativ kein größerer Blutverlust mehr beobachtet; der unmittelbar vor Schnitt kontrollierte Hb-Wert lag bei 11,7 g/dl, die unmittelbar postoperative Kontrolle bei 9,7 g/dl und am Morgen des Folgetages bei 8,1 g/dl. Der Base Excess (BE) als Schockindikator lag postoperativ bei 0,8 mmol/l und nach weiteren 90 min bei 0,9 mmol/l. Der intraoperative Blutverlust wurde mit 700 ml dokumentiert; auf die Transfusion von Erythrozytenkonzentraten konnte im Kontext der Akutversorgung des Patienten sowie innerhalb der ersten 24 h verzichtet werden. Die konventionellen Gerinnungsparameter lagen für den INR unmittelbar postoperativ bei 1,01, PTT 34,4 s, und die Thrombozyten fielen zu keinem Zeitpunkt innerhalb der ersten 24 h ohne Substitution unter 110/nl. Bei Dissektion der linken Arteria iliaca communis wurde bereits 4 h nach der Operation mit einer prophylaktischen Antikoagulation mit 400 IE/h Heparin begonnen. Darunter lag die PTT in den ersten 24 h immer zwischen 30 und 36 s; thrombembolische Komplikationen entwickelten sich in der Akutphase nicht.

## Diskussion

Der Fall zeigt die notfallmäßige Behandlung eines Schwerstmehrfachverletzten (Injury Severity Score (ISS) 66) mit lebensbedrohlicher traumatischer intraabdomineller Blutung unter Dauertherapie mit dem Faktor-Xa-Hemmer Apixaban und verbliebener gerinnungsrelevanter Restaktivität durch zeitnahe effektive Antagonisierung mittels Andexanet alfa, „Point-of-care“-gesteuerter viskoelastizitätsbasierter Gerinnungstherapie und chirurgischer Blutungskontrolle.

Laut Daten des TraumaRegister der Deutschen Gesellschaft für Unfallchirurgie (TR-DGU®) wurde 2019 fast jeder 4. Schwerstverletzte (*n* = 6171; 24,1 %) direkt zur (Notfall)Operation in den OP weiterverlegt [1]; 20 % (*n* = 4452) davon hatten eine vorbestehende Störung ihrer Gerinnungsfunktion, und wiederum 20 % (*n* = 2569; Daten im reduzierten TR-DGU®-Datensatz nicht verfügbar) benötigten eine medikamentöse Hämostasetherapie. Seit 2019 steht Andexanet alfa zur Anwendung bei Erwachsenen zur Verfügung, die mit den direkten FaktorXa(FXa)-Inhibitoren Apixaban oder Rivaroxaban behandelt werden, wenn aufgrund lebensbedrohlicher/nichtkontrollierbarer Blutungen eine Aufhebung der Antikoagulation erforderlich ist. Aufgrund fehlender Daten wird die Anwendung zur Aufhebung des FXa-Inhibitors Edoxaban aktuell in Deutschland nicht empfohlen, obgleich die Substanz in Japan hierfür zugelassen ist [2]. Das empfohlene Dosierungsschema orientiert sich an der zum Zeitpunkt der Aufhebung eingenommenen Apixabandosis sowie der Zeit nach letztmaliger Einnahme [2]. Im vorliegenden Fall erfolgte eine niedrigdosierte Therapie mit Andexanet alfa bei eigenanamnestischer Dauermedikation mit Apixaban 2,5 g 2‑mal täglich und letztmaliger Einnahme am Morgen des Unfalls.

Erleichtert wurde die Therapieentscheidung durch die eigenanamnestische Angabe des am Unfallort wachen Patienten in Bezug auf die Dauereinnahme von Apixaban infolge einer stattgehabten zentralen Lungenarterienembolie. In Zusammenschau von sowohl prä- als auch intrahospital ultrasonographisch nachweisbarer intraabdomineller Flüssigkeit und dem klinischen Bild eines zunehmenden hämorrhagischen Schocks wurde frühzeitig eine erweiterte viskoelastizitätsbasierte Diagnostik mittels ClotPro® einschließlich der differenzialdiagnostischen Untertests auf DOAK-Einnahme eingeleitet. Während über den ClotPro®-ECA-Test (Ecarintest) der zeitnahe Nachweis von direkten Thrombinhemmern erfolgt, besitzt der hier genutzte RVV-Test eine hohe Sensitivität für Faktor-Xa-Hemmer. In einer Kohortenstudie an 108 Traumapatienten mit Vorhofflimmern unter DOAK-Therapie korrelierten beide Tests mit den parallel detektierten Plasmaspiegeln [3]. Im vorliegenden Fall zeigte der RVV-Test eine deutlich verlängerte CT-Zeit und somit eine noch deutlich vorhandene und gerinnungsrelevante Restaktivität des am Morgen eingenommenen Faktor-Xa-Hemmers Apixaban.

In den Zulassungsstudien wurde gezeigt, dass Andexanet alfa die Wirkung von FXa-Inhibitoren innerhalb von Minuten aufhebt [4]. In der im Intervall durchgeführten ClotPro®-RVV-Kontrolle zeigte sich eine rasche Normalisierung der CT-Zeit und somit eine effektive Antagonisierung. Aktuell existiert keine alternative laborchemische Methode, die die Überwachung einer Behandlung mit Andexanet alfa ermöglichen würde. Insbesondere die Messung der Anti-FXa-Aktivität mit kommerziellen Methoden ist ungeeignet, da diese die Aufhebungsaktivität von Andexanet alfa unterschätzen. Der Hersteller empfiehlt daher, die Überwachung des Therapieerfolges anhand der klinischen Wiederherstellung der Hämostase und des Nichtauftretens von thrombembolischen Ereignissen durchzuführen [5].

Bereits gegen Ende des operativen Eingriffs imponierte eine wiederhergestellte und regelhafte Hämostase mit normalisierten Gerinnungsparametern in der laborchemischen Kontrolle. Der intraoperative Blutverlust wurde mit 700 ml dokumentiert; auf die Gabe von Erythrozytenkonzentraten konnte im Rahmen des Notfallmanagements komplett verzichtet werden. Bei diagnostizierter Dissektion der linken Arteria iliaca communis erfolgte nach Kontrolle von Blutung und Wiederherstellung der Hämostase die frühzeitige Antikoagulation. Eine Interaktion zwischen Andexanet alfa und der Heparinwirkung wurde nicht beobachtet.

## Fazit für die Praxis

1. Die Anzahl Schwerstverletzter und blutender Patienten unter DOAK-Dauertherapie wird weiter zunehmen.
2. Weiterentwickelte viskoelastische Testverfahren haben die Diagnostik in der Notfallsituation verbessert.
3. Spezifische Antidote stehen zur Verfügung.
4. Traumazentren sollten die Möglichkeit zur notfallmäßigen Antagonisierung vorhalten.
